# Shape, Size, and Robustness: Feasible Regions in the Parameter Space of Biochemical Networks

**DOI:** 10.1371/journal.pcbi.1000256

**Published:** 2009-01-02

**Authors:** Adel Dayarian, Madalena Chaves, Eduardo D. Sontag, Anirvan M. Sengupta

**Affiliations:** 1 Department of Physics and Astronomy, Rutgers University, Piscataway, New Jersey, United States of America; 2 Project COMORE, INRIA, Sophia-Antipolis, France; 3 Department of Mathematics, Rutgers University, Piscataway, New Jersey, United States of America; 4 BioMaPS Institute for Quantitative Biology, Rutgers University, Piscataway, New Jersey, United States of America; Lawrence Berkeley National Laboratory, United States of America

## Abstract

The concept of robustness of regulatory networks has received much attention in the last decade. One measure of robustness has been associated with the volume of the feasible region, namely, the region in the parameter space in which the system is functional. In this paper, we show that, in addition to volume, the geometry of this region has important consequences for the robustness and the fragility of a network. We develop an approximation within which we could algebraically specify the feasible region. We analyze the segment polarity gene network to illustrate our approach. The study of random walks in the parameter space and how they exit the feasible region provide us with a rich perspective on the different modes of failure of this network model. In particular, we found that, between two alternative ways of activating Wingless, one is more robust than the other. Our method provides a more complete measure of robustness to parameter variation. As a general modeling strategy, our approach is an interesting alternative to Boolean representation of biochemical networks.

## Author Summary

Developing models with a large number of parameters for describing the dynamics of a biochemical network is a common exercise today. The dependence of predictions of such a network model on the choice of parameters is important to understand for two reasons. For the purpose of fitting biological data and making predictions, we need to know which combinations of parameters are strongly constrained by observations and also which combinations seriously affect a particular prediction. In addition, we expect naturally evolved networks to be somewhat robust to parameter changes. If the functioning of the network requires fine-tuning in many parameters, then mutations causing changes in regulatory interactions could quickly make the network dysfunctional. For predictions involving gene products being ON or OFF, we found a method that facilitates the study parameter dependence. As an example, we analyzed several competing models of the segment polarity network in *Drosophila*. We explicitly describe the region in the parameter space where the wild-type expression pattern of key genes becomes feasible for each model. We also study how random walks in the parameter space exit from the feasible region of a network model, allowing us to compare the relative robustness of the alternative models.

## Introduction

Robustness, in the context of biological networks, broadly indicates that the system remains viable under different perturbations. Defining robustness in a precise form is a challenging task, given that robustness to different kinds of perturbations, e.g., environmental variation, intrinsic fluctuations in chemical networks or changes due to mutations, might involve different features of an existing network [1],[2]. In this paper, we are concerned with the robustness of functionality to changes in the kinetic parameters for a given network architecture. In an influential study of the Drosophila segment polarity network, robustness has been associated to the fractional volume of the region in parameter space associated with the wild type gene expression pattern [3]. In this paper we will see that the geometry of the space of feasible parameters contains additional information on essential aspects of robustness and fragility of the network.

In the context of fitting biochemical kinetics models to time series data, investigators have looked at effects of small parametric perturbations on the quality of the fit. Sensitivity analysis [4],[5], namely considering the effect of changing parameters, one at a time, is a common practice by now. Brown and Sethna have looked at correlated changes of parameters and study how moving in different directions in parameter space affects the predictions [6]. Based on the eigenvalues and the eigenvectors of the Hessian of the cost function at the minimum, these authors and their collaborators find that, for many known biochemical networks, only a few directions in the parameter space have stiff constraints whereas the rest of the directions are “sloppy” [7],[8]. In this work, we will consider the segment polarity network as an example and will explicitly characterize the region in parameter space where the network could be functional. The anisotropy in the shape of this feasible region will become apparent from our analysis. We should clarify that the robustness of a model to parameter variation, as measured by goodness of fit to data, is distinct from the robustness of the system functionality with respect to parameter variation from mutations. However, at a mathematical level, these two problems just give rise to different ways of scoring parameter choices for a model, and there is much that is parallel in the consideration of the shape of the regions that score well in each of these problems.

The segment polarity network is part of a cascade of gene families responsible for generating the segmentation of the fruit fly embryo. Genes involved in initiating this pattern are transiently expressed, and interactions among the segment polarity genes should maintain and fine-tune this pattern as the embryo grows through cell division. Much of the information about this network comes from genetic analysis and are therefore of qualitative nature. In particular, we do not know many of the parameters necessary to describe this dynamical system. This is a common situation faced in modeling most biochemical networks.

In their work on modeling the segment polarity network, von Dassow et al. [3] encountered the same problem. Their approach was to solve an ODE model of the network for random choices of parameters and then score the resultant expression patterns based on compatibility with the experimentally observed wild type pattern. If this score is found to be above a certain threshold, the given parameter combination is said to belong to the feasible region of the parameter space. Robustness of a particular architecture is then ascertained by the fractional volume of the feasible region, estimated from their simulation. Ingolia [9] looked at a set of criteria for bistability in particular submodules of the network and studied the extent to which these criteria describe this feasible region. In general, providing an approximate description of the structure of feasible region, even for a medium size biochemical network, remains an important challenge.

One could also get some insight into the functioning of the network by constructing a model where each gene or gene product is mostly ON or OFF. For example, in the context of this particular network, Boolean models have been employed to study dependence upon initial state or the effect of deletion of particular components [10]. Unfortunately, addressing questions related to parameter dependence is not possible within the conventional Boolean framework. Therefore, we develop a new approximation, within which the treatment of our model shares the simplicity of Boolean analysis without sacrificing the possibility of exploring parameter dependence issues. This approximation enables us to explicitly characterize the feasible region in the parameter space of the model.

If a point in the feasible region of parameters represents a functional biological system, then a mutation causes the system to jump to a new point. If this new point also belongs to the feasible region, the system is robust with respect to that mutation. Otherwise the mutation is deleterious. If the jump in the parameter space, caused by a mutation, is relatively large then the result of successive mutations is to quickly probe different regions of the parameter space. In this case, robustness essentially depends on the volume of the feasible region. On the other hand, if the jumps in the parameter space are relatively small, evolution of parameters due to successive mutations can be represented by a random walk in the parameter space. The idea of representing evolution as a continuous random process has already been used in the adaptive landscape approach [11]. In this case, the random walk exiting the feasible region in the parameter space corresponds to a deleterious mutation. The exit time distribution is very sensitive to the shape of the feasible region. Robustness to mutation is, now, related to the features of this distribution (e.g. half-life, asymptotic decay rate, etc.) [12] and therefore depends upon the shape and not just the volume of the feasible region.

If we want to choose a single measure for robustness, the inverse of the asymptotic decay rate is a good candidate [12]. This measure is sensitive to the geometry (both volume and shape) of the feasible region. For example, even if the total volume of the feasible region is relatively large, existence of “narrow” directions will greatly affect the decay rate; or if the feasible region is constituted of several disconnected part, the decay rate will again be affected. In addition, it is independent of the initial condition. Also, in the theoretical case, where every mutation leads to a new, uncorrelated point in the parameter space, the inverse of the asymptotic decay rate is a simple function of the fractional volume of the feasible region.

In our study, we will estimate half-life, a different but closely related measure of robustness. In case a single exponential in time gave the probability of remaining in the feasible region, these two measures of robustness would be proportional to each other. In practice, half-life depends partially on short time properties of the system and is initial condition dependent. On the other hand, measuring the asymptotic decay rate accurately for high dimensional stochastic system needs more computational effort than estimating half-life.

Before we go on, let us explain what measure of distance we use when we talk about narrow or wide directions in the parameter space. If we consider the continuous random walk approximation to parameter evolution, then the short-time properties of diffusion set up a metric for the space of parameters. The metric tensor of this space is the inverse of the covariance matrix of infinitesimal displacements divided by the infinitesimal time interval. Once we have this metric, we could decide whether, from a generic point, the distance to reach the boundary in certain direction is relatively small or large. This definition of distance is closely tied to the time the system typically takes to diffuse over a certain separation.

Once we characterize the feasible region in parameter space, we explore how the system fails as a result of such a random walk. For two alternative network models, we compare the exit time distributions. More importantly, we can see how, in a particular model, the feasible region is narrower in certain directions than in others. These narrow directions are associated with the predominant modes of failure of the system in the random walk process. We end by speculating how these methods could be extended to generic biochemical network models.

## Results

**Figure 1:**
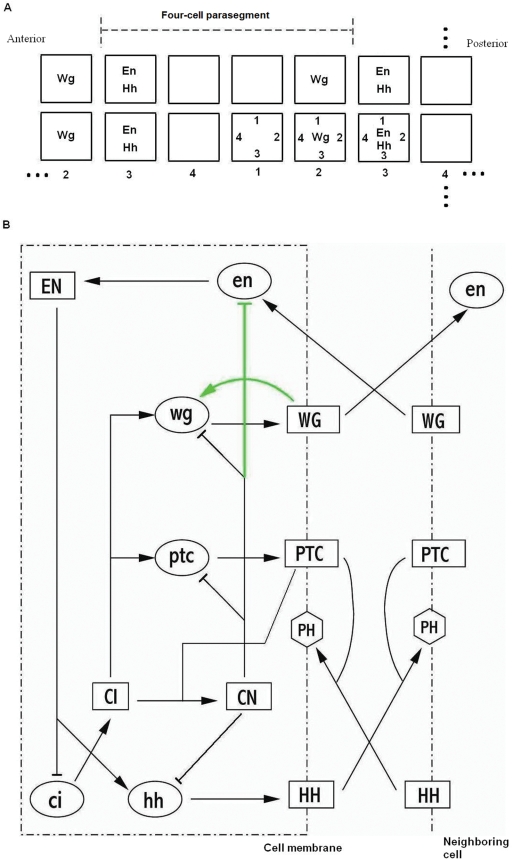
Expression pattern for key segment polarity genes and the interaction network. (A) Four cells in a parasegment with periodic boundary conditions in both dimensions. Each cell is represented by a square. The convention for numbering cells and cell faces are shown. (B) Interaction network used in reference [3]. Two green lines indicate interactions added by authors to achieve the target pattern. Black lines indicate interactions based on experimental data. Shape of the nodes indicates the corresponding component: Ellipses represent mRNAs; rectangles, proteins.

In the wild type segment polarity pattern, genes are expressed periodically in 14 parasegments along the fly embryo, and each parasegment consists of four stripes of cells. Because of this periodicity, one could focus only on one parasegment or in other words only on 4 cells. Figure 1A shows the wild type gene expression pattern for three key components of the segment polarity network. For simplicity, each cell is assumed to have four faces, rather than six as in the original model [3]. When using abbreviated names for components of the network, we use uppercase letters to refer to proteins and lowercase letters for the corresponding mRNAs. *Wingless* (*WG*) is a signaling molecule known experimentally to activate *Engrailed* (*EN*) through cell-to-cell communication. *EN*, itself a transcription factor, in turn triggers the production of another signaling molecule, *Hedgehog* (*HH*). *HH* then gets secreted to the neighboring cell and maintains *WG* expression by stabilizing an activator of *wg*, called *Cubitus interruptus* (*CI*). Without *HH* signaling, *CI* gets proteolytically cleaved, leaving only its amino terminus (denoted by *CN*), which becomes a repressor of *wg*. In summary, experimentally it is known that *WG* and *EN* maintain the expression of each other through cell-to-cell communication. We represent the wild type expression pattern of these mRNA components as follows:
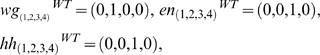
(1)where the four entries of each of the vectors correspond to the gene expression in the four cells of a parasegment. The value “0” means the gene is turned off and the value “1” means it is maximally expressed.

The abovementioned mechanisms leave room for the following questions. Why is *EN* expressed only posterior to the *WG* expressing stripe? The anterior cell also receives a *WG* signal but does not produce *EN*. Similarly, one could ask why *WG* is expressed only anterior to the *EN* expressing stripe.

Figure 1B shows the interaction network used in reference [3]. In that work, the authors started only with interactions shown by black lines but were unable to reproduce the right pattern in their simulations. The best pattern authors could achieve, using only black lines, was an alternative expression of *wg* and *en* in all cells. Therefore, authors decided to add two new interactions shown with green lines. With these links in place, they were able to find many parameter combinations to reproduce the target pattern.

To explore the dependence of robustness of the network on its topology, Albert and Othmer [10] developed a Boolean model of the segment polarity network, a discrete logical model where each species has only two states (OFF or ON), but no kinetic parameters need to be defined. This Boolean model is amenable to various methods for systematic robustness analysis [10], [13]–[15]. Unfortunately, the ease of analysis comes at the cost of not being able to address questions related to the parameter dependence.

### A Step Function Approach to the Segment Polarity Network Model

We propose an approach which retains the information about kinetic parameters, but, at the same time, keeps part of the simplicity of a Boolean model by having most genes either in the fully ON or the fully OFF state. We approach the problem by first solving the algebraic equations coming from the steady state conditions and writing the steady state solutions in terms of the parameters. Since one of the steady state solutions should match the wild type pattern, one can look for the constraints on parameters that yield this pattern. This procedure provides a family of conditions defining regions of feasible parameters for the wild type steady state. Although all of the parameters in the feasible region can maintain the desired pattern, one aspect we ignore is whether the system can reach the wild type pattern from particular initial conditions.

In our analysis, we used the fact that many of the differential equations in the model involve terms of the Hill form:
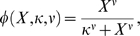
where *X* is the concentration of some species, *κ* is the dissociation constant and *ν* is the Hill coefficient. The steepness of the Hill function is characterized by the Hill coefficient *ν*. As *X* increases from zero and passes the threshold *κ*, the function *φ* has a transition from OFF to ON state. For moderately large Hill coefficient, this transition becomes quite steep, and *φ* is practically insensitive to the actual value of *ν*. In the model presented in reference [3], *ν* is indeed found to be often quite large, between 5 to 10 [16]. Any such term may thus be replaced by a step function with two levels:
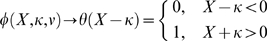



Using this, the steady state gene expression is characterized by the following equations:
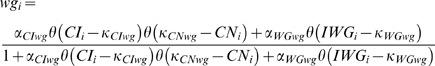
(2)

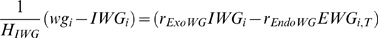
(3)

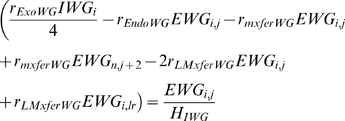
(4)

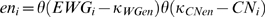
(5)

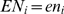
(6)

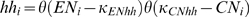
(7)

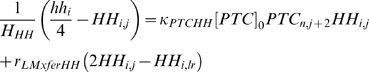
(8)

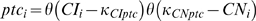
(9)

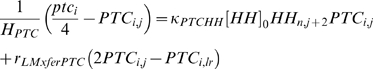
(10)

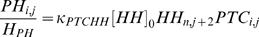
(11)

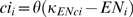
(12)

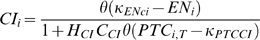
(13)

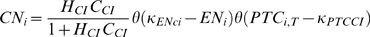
(14)


Here we use the same notation as in [3]. *X_i_*, *i* = 1,2,3,4, denotes the total concentration of the protein species *X* in cell *i,* with lower case *x_i_* referring to the concentration of the corresponding mRNA molecules. In addition, for three of the components involved in cell-to-cell communication, namely, external *Wingless* (*EWG*), *Patched* (*PTC*) and *HH*, the concentration on each of the four cell faces could be different. For any of these components, the concentration in cell *i* at face *j* is denoted by *X_i_*_,*j*_, *i* = 1,2,3,4, *j* = 1,2,3,4. For these three species, the sum of the concentration over all four faces of cell *i* is denoted by *X_i,T_*. The adjacent cell face to face *j* of cell *i* is shown by *X_i,lr_*. The opposite cell face to face *j* of cell *i* is shown by *X_n,j_*_+2_.

Also, *κ_XY_* denotes the dissociation constant for species *Y* corresponding to the binding that regulates the species *X*_._ The range for *κ_XY_* is chosen to be between zero and one. The equations are in normalized form, meaning that the concentrations of the components have been scaled so that the maximal steady state level is one.

The structure of this particular network allows one to draw several interesting conclusions immediately. For example, the steady state levels for *HH* and *PTC* are completely determined once one specifies the mRNA levels of *en*, *hh* and *ptc* (this does not depend on the high Hill coefficient approximation). Assuming that *en* and *hh* are expressed only in the cell 3, which is the case in the wild type pattern, it can be shown that *ptc*_2_ = *ptc*_4_, and *PTC*_2,*T*_ = *PTC*_4,*T*_. The reason is as follows. If *ptc*_2_>*ptc*_4_, cell 2 ends up producing more *PTC*, part of which get bound to *HH* diffusing over from cell 3. However, the symmetric nature of the diffusion leads to more *PTC* in cell 2 than in cell 4: *PTC*_2,*T*_>*PTC*_4,*T*_. Higher level of *PTC* results in higher rate of proteolysis of *CI*. Therefore, in the steady state, *CI_i_* is a decreasing function of *PTC_i_* and *CN_i_* is an increasing function of *PTC_i_*. This means that (given *en* is not present in cells 2 and 4, and therefore has no repressive effect on *ci* production)
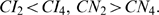
(15)


However *CI* is an activator and *CN* is a repressor of *ptc*, which together with Equation 15 implies *ptc*_2_<*ptc*_4_, which contradicts the assumption we started with. Of course, we could have started with *ptc*_2_<*ptc*_4_ and again end up with contradiction (for the formal proof, see, Chaves, Sengupta and Sontag, Geometry and topology of parameter space: investigating measures of robustness in regulatory networks, to appear in Journal Mathematical Biology). This argument shows that the concentration levels of *ptc*, *PTC*, *CI*, *CN* and *PH* is exactly the same in cells 2 and 4:
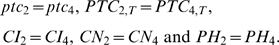
(16)


This observation will turn out to be quite significant for the following reason. The *wg* level in a cell is controlled by the *CI-CN* pathway and the postulated feedback [3] from internal *WG* (*IWG*). Since cells 2 and 4 do not differ when it comes to *CI* and *CN* levels, any difference in the *WG* expression has to be attributed to the *wg* autoregulation.

In order to analyze the *wg* sector, we note that, in this model, the *EWG* and *IWG* levels are uniquely determined by a set of linear equations once the *wg* levels are given. Solving these linear equations, using the periodic boundary conditions and the fact that *wg* is produced only in cell 2, we find that:
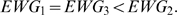
(17)


This result is not surprising because the distribution of *WG* is determined by a symmetric diffusion process from the source in cell 2, the only *wg* producing cell in each parasegment. Therefore, we expect cells 1 and 3 to have identical amounts of *WG* signaling. It turns out that *EWG* at the source, cell 2, is higher than that of the flanking cells (the formal proof is presented in the supplementary material). These observations have important consequences for the regulation of *en*, as explained below.

Since *en* is expressed in cell 3, we have:
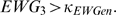
(18)


This, together with Equation 17, implies:
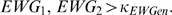
(19)


Had the *en* production been solely controlled by *EWG*, the model would have implied that if *EWG*_3_ is high enough to activate *en* in cell 3, *en* will be also activated in cells 1 and 2. This is why, in reference [3], adding repression of *en* by *CN* was necessary to achieve the wild type expression pattern. The two new links introduced in reference [3] (green lines in Figure 1B) give rise to two positive feedback loops. The *wg* autoactivation gives rise to bistability, allowing cells 2 and 4 to have distinct levels of *wg* expression. The other loop (*En* ^__^| ci→*CI*→*CN* ^__^| *en*→*EN*), generated by adding repression of *en* by *CN*, is required to prevent *en* from being expressed in cells 1 and 2. This also requires *CN* to be expressed in those cells. The bistability of the *EN*-*CI*-*CN* system allows cells 1 and 3 to have different *en* level even when the external *Wg* signal is the same for both of them.

We should note that autoactivation as a way for maintaining the *WG* expression is problematic in the following sense. In the model described above, *wg* is always activated via autoactivation and the preexisted *CI-CN* pathway never contributes to the pattern. This is in contrast with the experimental data, which suggests that *HH* signaling from the neighboring cell plays a crucial role in maintaining the *wg* expression. The fact that model [3] does not depend upon *HH* signaling for maintaining the expression of *wg* manifests itself when cell division is considered. In this model, both daughters of a cell in the *wg*-expressing stripe are able to retain the *wg* ON state through autoactivation. This causes the stripe to grow wider and wider over cell divisions. However, in wild type fly, the *wg*-expressing stripe should remain one cell wide. The daughter cell, which is further from the *en*-expressing stripe, and therefore not exposed to *HH* signaling, loses *wg* expression. This means that one stripe of *WG* is left after each division. Ingolia [9] has also noticed that in this model, *IWG* level must always be above K*_WGwg_* (the autoactivation threshold) in the cell that expresses *wg*. When we removed the *CI*-*CN* cycle for activation of *wg* from the simulation performed in reference [3], the fraction of “good solutions” increased by a factor of 3. This suggests that most of the time the *CI-CN* pathway is either not contributing to *WG* expression or it leads to misexpression of *WG* in cell 4.

The model is too dependent on the bistability of the two sub-networks with positive feedback for maintaining four cell expression patterns. One could avoid this problem by making some of the four cells special, either by inclusion of other genes in the network or by explicitly breaking the symmetry via introducing different gene expression rates from cell to cell for some of the genes already in the model.

The major candidate for inclusion in the model is the *Sloppy-paired* protein (*SLP*) as has already been suggested by others [9],[10],[17]. *SLP* is only present in cells 1 and 2: 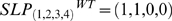. It is a necessary (but not sufficient) factor for activation of *wg* and it also represses *en*. In the presence of *SLP*, the reason *en* is not expressed in cell 1 despite *WG* signaling is that it is being repressed by *SLP*. Also, despite *HH* signaling, *wg* is not produced in cell 4 because *SLP* is not present there. With *SLP* added, the two new interactions introduced in [3] are not necessary anymore, and also *WG* expression will depend on the *CI-CN* pathway.

In this paper, we will analyze the effect of including *SLP*. We keep *SLP* as an external factor meaning that the expression pattern of *SLP* is given. However, it can easily be incorporated into the network. If *WG* activates *SLP*, a positive feedback loop is formed which allows for bistability: both *WG* and *SLP* can be ON or both can be OFF. On the other hand, if *EN* represses *SLP*, another positive feedback loop is formed which again allows for bistability: *SLP* can be ON and *en* OFF or vice versa. We have also explored a model with explicitly different rates of production of *ptc* and *ci* from cell to cell which will be presented in a separate publication (Chaves, Sengupta and Sontag, Geometry and topology of parameter space: investigating measures of robustness in regulatory networks, to appear in *Journal Mathematical Biology*). The work also presents a study complimentary to that presented in this paper. It provides an explicit geometric description of the feasible region by partitioning the region into components defined by algebraic inequalities, in other words, by constructing a cylindrical algebraic decomposition.

**Figure 2:**
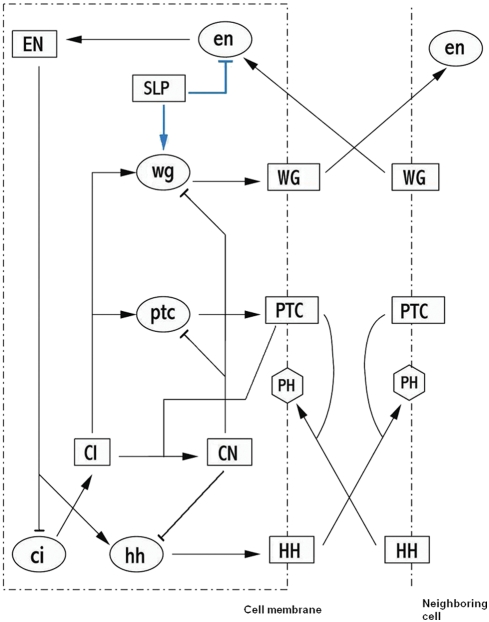
Segment polarity regulatory network including *sloppy-paired* protein. In this model, the possibility of *Wg* autoactivation and *en* repression by *CN* is not included.

Here, we consider two particular cases:

1. The regulatory network used by von Dassow et al. [3]. This network is shown in Figure 2B. We will refer to this case as von Dassow et al. model.
2. The regulatory network including *Sloppy-paired* protein, but without the two positive feedback links introduced in [3]. This network is shown in Figure 2. We will refer to this case as *SLP* model.

We can explicitly write down the conditions characterizing the feasible region for these two models. The results are presented in Tables 1 and 2 (see Materials and Methods for the derivation of these conditions). We could easily estimate the associated volume of feasible region by randomly choosing points in the parameter space and check whether they satisfy the appropriate conditions. As we discussed in the introduction, the fate of random walks, especially where they exit the feasible region, teaches us a lot about relative vulnerability of different constraints.

**Table 1:** Conditions characterizing the feasible region for the regulatory network used by von Dassow and collaborators.

Condition Number	Condition
**1**	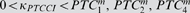
**2**	1>*κ_CIwg_*>1−*Z_C_* or 0<*κ_CNwg_<Z_C_*
	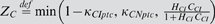
**3**	0<*κ_EWGen_*<*EWG*_3_
**4**	0<*κ_CNen_*<*Z_C_*
**5**	max{*IWG*_1,3,4_}<*κ_WGwg_*<*IWG*_2_

This network, shown in Figure 1B, includes two positive feedback loops achieved by adding *WG* autoactivation and *en* repression by *CN*.

**Table 2:** Conditions characterizing the feasible region for the regulatory network including *Sloppy-paired* protein.

Condition Number	Condition
**1**	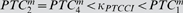
**2**	(1>*κ_CIwg_*>1−*Z_C_* and 0<*κ_CNwg_*<1) or (1>*κ_CIwg_*>0 and 0<*κ_CNwg_*<*Z_C_*) 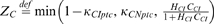
**3**	*EWG*_4_<*κ_EWGen_*<*EWG*_3_

In this network, shown in Figure 2, the two links of *WG* autoactivation and *en* repression by *CN* are absent.

### Random Walk in the Feasible Region

**Figure 3:**
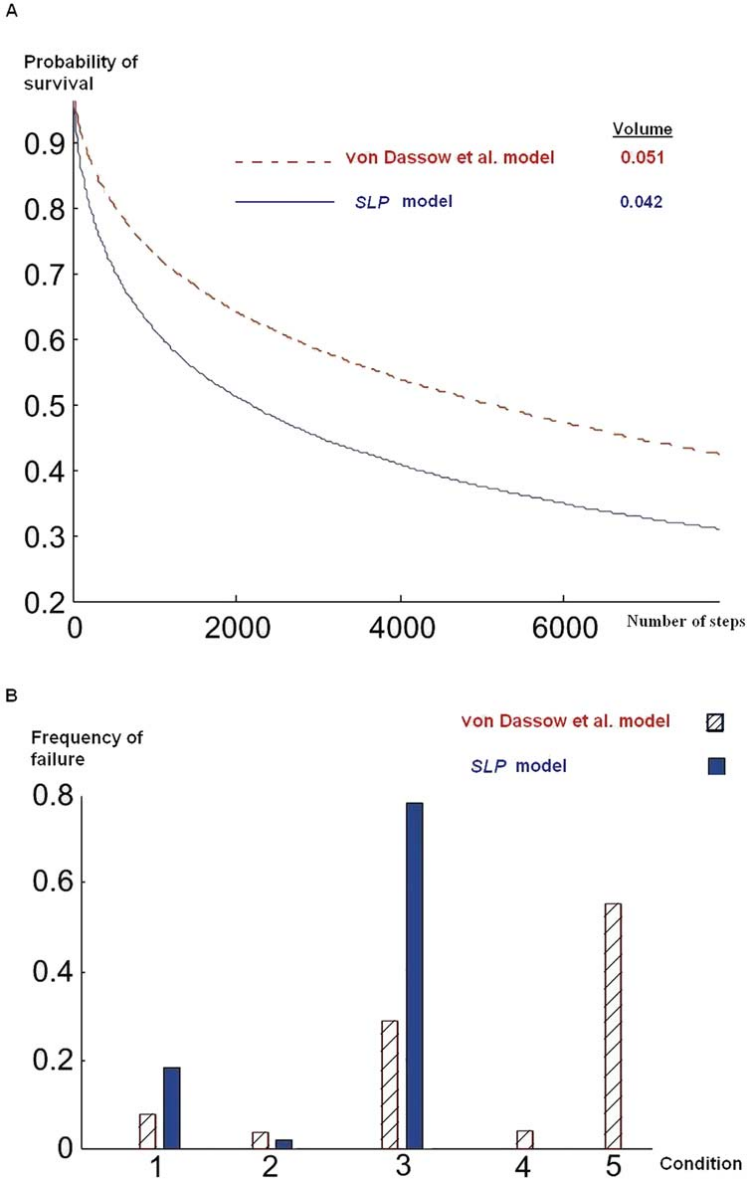
Random walk in the space of admissible parameters. We choose a random point from admissible parameter set and follow a random walk until it hits a boundary after *t* steps. (A) The red (and dashed) and the blue (and solid) graphs represent the probability of survival as a function of time for von Dassow et al. and *SLP* models, respectively. These graphs results from 30,000 runs of random walks. The results given for volume are based on the fraction of feasible parameter combinations found in 1,000,000 randomly chosen combinations. (B) Histogram of violated conditions for the random walk in (A). The number above each bin indicates the corresponding condition in Tables 1 and 2.

We explore the feasible region by following random walks starting from random points. Whenever one of the random trajectories hits a boundary and exits the feasible region, we terminate the walk and keep track of the inequality that was violated. This process can be viewed as a simulation of parameter evolution due to mutations in a fitness landscape that looks like a plateau. The points in the feasible region have a constant high fitness, and the rest of the points have zero fitness. The result of the simulation is presented in Figure 3.

For the two models discussed, the graphs in Figure 3A show the probability of survival as a function of time. This is the probability that the random walk has not exited the feasible region in the first *t* steps. From the graph, we can easily measure *T*_1/2_, defined as the time for which there is a 50% chance that the system has already suffered a deleterious mutation. As we discussed in the introduction, this number is a possible indicator of robustness.

Figure 3B shows the histogram of violated conditions. The number below each bin indicates the corresponding condition in Tables 1 and 2. The lead cause of failure in the von Dassow et al. model is the constraint on *κ_WGwg_* whereas in the *SLP* model it is the constraints on *κ_EWGen_*. Higher vulnerability of the *SLP* model with respect to the constraint on *κ_EWGen_* can be understood by comparing condition 3 in Table 1 and the corresponding condition in Table 2. In the *SLP* model there is a lower bound on *κ_EWGen_* coming from the fact that *κ_EWGen_* should be greater than *EWG*_4_ to prevent activation of *en* in cell 4. However in the von Dassow et al. model, *en* is being repressed by *CN* and therefore there is no lower limit on *κ_EWGen_*.

One might raise the question of whether including repression of *en* by *CN* in the *SLP* model changes the constraints on *κ_EWGen_*. In high Hill coefficient limit, adding this interaction does not change the conditions in Table 2. To see this, notice that as was mentioned before, requiring *CI* and *CN* levels to be different in cells 1 and 2 forces us to have *CN*_2_ = *CN*_4_ = 0. In cell 4, *CN* is not expressed, and in cells 1 and 2, *en* is already being repressed by *SLP*. Therefore, adding the possibility of *en* repression by *CN* does not change any of the constraints.

If we consider the case where Hill coefficients in the *CI*-*CN*-*PTC* sector are small, the transition from high to low in concentration value for *ptc*-nullcline and *CN*-nullcline would not be sharp. Instead, the transition would happen over a wide range. This means that we would get a non zero value for *CN*_4_. In that case, adding repression of *en* by *CN* can indeed help in maintaining the wild type pattern, thereby increasing the robustness of the model.

The parameters *κ_CIwg_*, *κ_CNwg_* and *κ_WGwg_* are related to alternative routes controlling *wg* expression. The first two parameters play an important role in deciding *WG* expression in the *SLP* model, while this role is played by *κ_WGwg_* in the von Dassow et al. model. Comparison of the frequency of failure for conditions 2 and 5 in the histogram in Figure 3B suggests that controlling *wg* via the *CI*-*CN* pathway in the presence of *SLP* is the more robust way of achieving the target gene expression pattern for *wg*.

What about adding the *WG* autoactivation to the *SLP* model? If one just cares about producing the right four-cell pattern for *en*, *hh* and *wg*, then this addition could give rise to more solutions. However, as we discussed before, not having *wg* production to be sensitive to *HH* signaling from the neighboring cell is problematic and gives rise to wide stripes of *wg* expression under cell division. If we constrain the model so that *wg* is sensitive to *HH* signaling via *CI*-*CN* pathway, we find that adding *wg* autoactivation to a functional solution in the *SLP* model often leads to misexpression of *wg* in cell 1 or cell 3, thereby shrinking the feasible region in parameter space.

## Discussion

Our results imply that the lack of robustness is not only dependent upon the size of the feasible region, but also upon the existence of critical directions along which this region is globally very narrow. We found relatively few constraints on the parameters given that we have specified the gene expression patterns for *en*, *hh* and *wg* in each of the four cells. Much has been said about the relation between the topology of the network and robustness. In practice, we found that it is not only the structure of the network but also the nature of the wild type expression pattern which plays an important role in the ultimate simplicity of the constraints that dictate robustness. For example, the fact that only one cell is expressing *en* and *hh* and that *wg* had no direct effect on the *CI*-*CN*-*PTC* sector allowed us to draw several conclusions about certain variables being the same in cell 2 and cell 4. If one only pays attention to the network structure, *wg* indeed has an effect on the *CI*-*CN*-*PTC* sector via its effect on *en*. However, specifying the *en* expression pattern hides the influence of *wg* and helps us disentangle the constraints. The role of *wg* shows up only when one insists upon self-consistency, namely, the *wg* expression pattern is going to lead to the target *en* expression pattern. Simplicity of the final constraints is not a result of some obvious modularity in the network itself but some combination of the network structure as well as of the sparseness of the expression pattern. We cannot be sure that this is a general feature of robust genetic networks. A broader study, which takes into account the role of the wild type pattern on the robustness of a network, would be a welcome deviation from discussions centered purely on network architecture.

We noted that capturing the *CI*-*CN*-*PTC* negative feedback in the Boolean model is difficult. For example, in the Boolean model constructed by Albert and Othmer [10], they are forced into a situation where *ptc* mRNA is OFF but *PTC* protein is ON. This is achieved because of an exception made in *PTC* production rule, namely, *PTC* can continue to be in the ON state even if there is no *ptc*. Of course, this implausible rule results in a distribution of *ptc* and *ci* products which mimics the wild type pattern. For example cell 1 has less *ptc* but more *CN* compared to cell 2. In our model, we partially capture the effect of the feedback. We can indeed get the *ptc* levels to vary between cell 1 and cell 2. Unfortunately, we saw that in the high Hill coefficient model, producing different *CN* levels requires fine-tuning of the parameters. Therefore, we understand why von Dassow et al. find that setting the Hill coefficients in the *CI*-*CN*-*PTC* sector to be small enhances their chance of finding good solutions [16].

The present approach shows that, in addition to volume, the topology and geometry of the feasible region have important consequences for the robustness of a system. Of special interest is the structure of the boundary in the parameter space that separates between functional and non-functional systems. In the models studied here, it was possible to describe this boundary explicitly as a collection of constraints. For a generic biochemical network model with a scoring function it may not be feasible to explicitly write down the boundary surface corresponding to the threshold of functionality. However, one could generate a sampling of the boundary surface by following random walks in the parameter space until it hits the boundary of the functional region (decided by a threshold score). Instead of what we did in this study, we could slightly alter our strategy and let the walk be reflected off the boundary. In that process the same walk would hit many neighboring points on the boundary surface. If one generates a large enough sample of boundary points, one could use methods like manifold learning [18],[19] to approximately reconstruct the boundary.

Contrast this method to boundary reconstruction from uncorrelated random sampling. One could generate many points some of which are inside the region and many others that are outside. Indeed, many machine learning techniques for classification involve learning decision boundaries from such data. However, when the good region has a very small fractional volume and many of the randomly sampled points outside this region are far from the decision boundary, most of the sampled points have very little impact on boundary reconstruction. The uncorrelated nature of the sampling is useful for getting a good estimate for the fractional volume, but makes the process of mapping the geometry inefficient. It would be better to take advantage of one good solution to generate other good ones for the purpose of exploring local geometry.

Whether these approaches work for analyzing biologically motivated network models remains to be seen. For an arbitrary random network, with an equally arbitrary random choice of gene expression pattern, the feasible region could have a very complex structure and the methods outlined would not be particularly useful for characterizing it. The hope is that, for biologically relevant networks with wild type gene expression patterns, the feasible region may be quite simple, geometrically, and could be approximately described by the approaches suggested above.

To summarize, our analysis of the segment polarity network provides us with insights regarding the constraints that are crucial for functioning of the system. We showed how the system is particularly vulnerable to parametric perturbations in certain directions in the parameter space. We believe that the ideas developed here could be applied to other regulatory networks, to explore how the shape of feasible region in the parameter space contributes to its robustness. Hill terms appear often in models of biochemical networks. A simpler model, obtained by replacing these terms with step function, could be useful, because such a model enjoys some of the simplicity of the Boolean networks, while retaining many of the parameters of the original model.

## Materials and Methods

### Derivation of Conditions Characterizing the Feasible Region

Here we analyze two particular cases:

1. The regulatory network used by von Dassow et al. [3] which we refer to as von Dassow et al. model (Figure 2B).
2. The regulatory network including *Sloppy-paired* protein, but without the two positive feedback links introduced in [3]. We will refer to this case as *SLP* model (Figure 2).

We first focus on case I. Equations 2–14 characterize this network. The wild type expression pattern for *wg*, *en* and *hh* is given in Equation 1. Since *en* is only expressed in cell 3, *ci* and *ptc* are expressed in all cells except cell 3:
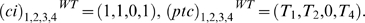
(20)


This is because in the absence of *EN*, *ci* is basally expressed which also leads to production of *ptc*. We will allow *T_i_* to take values between zero and one. The reason for the special, non-Boolean, treatment of *ptc* has to do with capturing the effect of the negative feedback loop in the *CI*-*CN*-*PTC* sector properly. This negative feedback loop leads to lower *ptc* level in cell 1 than in cells 2 and 4, as we shall see. The *ptc* level in cells 2 and 4 turn out to be comparable (*T*_2_ = *T*_4_). This is also the experimentally observed expression pattern of *ptc* [20].

**Figure 4:**
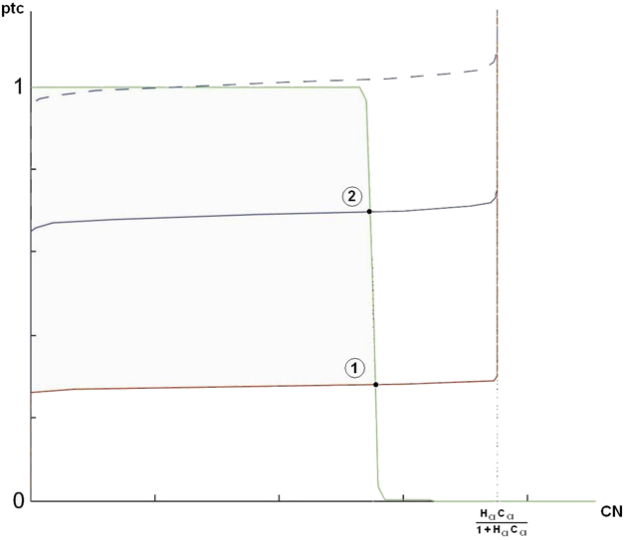
The nullclines for *ptc* and *CN*. The green curve shows the *ptc*-nullcline. In the high Hill coefficient limit, *ptc* value drops sharply from one to zero as *CN* passes the threshold of min(1−*κ_CIptc_*, *κ_CNptc_*). Blue and red curves show the *CN*-nullclines for relatively higher and lower values of *HH* signaling levels, respectively. Intersection points 1 and 2 determine *CI*, *CN* and *ptc* in cell 1 and 2/4, respectively. Here it is assumed that the negative feedback on *ptc* coming from repression by *CN* is active. Therefore, *ptc* and *CN* are not maximally expressed. Dashed blue line shows the *CN*-nullcline for a fine-tuned set of parameters.

How could we ever get such an intermediate values in our approach? First, from Equations 13 and 14, in the cells where *en* is not expressed and therefore *ci* is not repressed, namely in cells 1, 2 and 4, we have *CI*+*CN* = 1⇒*CI* = 1−*CN* (this does not depend on the high Hill coefficient approximation). Since *ptc* is regulated by *CI*-*CN*, we could draw one nullcline expressing *ptc* concentration as a function of *CN*. This curve is represented by the green graph in Figure 4. We will call it the *ptc*-nullcline. Here it is assumed that the negative feedback on *ptc* coming from repression by *CN* is active. This means that *CN* and *ptc* are not expressed maximally. For *ptc* to be expressed, the activation by *CI* requires 1−*CN*>*κ_CIptc_*⇒*CN*<1−*κ_CIptc_*. In addition, we need *CN* to be smaller than *κ_CNptc_* to avoid repression of *ptc* by *CN*. Thus, for values of *CN* smaller than the threshold of min(1−*κ_CIptc_*, *κ_CNptc_*), *ptc* is fully expressed. As *CN* passes this point, the value of *ptc* will drop sharply. In the high Hill coefficient limit, *ptc* will abruptly fall to zero.

On the other hand, *CN* production itself is dependent upon *PTC* protein. *PTC* is a monotonically increasing function of *ptc* and a decreasing function of *HH* signaling. Therefore, for a fixed value of *HH* level, we can also look at the concentration of *CN* as a function of *ptc*. This provides us with the *CN*-nullcline which depends upon the *HH* signaling strength. If we think of *CN* as a function of *ptc* level, the transition in *CN* from low level to its highest value happens at a particular *ptc* threshold, where the *PTC* level is just enough to start producing *CN*. If the cell is exposed to more *HH* signaling, sequestering away a larger fraction of total *Patched* protein, one needs more *ptc* to reach this threshold. The blue and the red graphs in Figure 4 show the *CN*-nullclines for relatively higher and lower values of *HH* signaling levels, respectively.

Because cell 1 receives less external *HH* signaling than cells 2 and 4, generally the red curve could be associated to cell 1 and the blue one to cells 2 and 4. The intersection points 1 and 2 determine *CI*, *CN* and *ptc* level in cell 1 and 2/4, respectively. As we see, *ptc* value could indeed be higher in cell 2 than in cell 1. However, *CN* concentration seems to be comparable in those cells. This is an artifact of our model where Hill coefficients are very large, which causes the transition from high to low in concentration value to happen in a very narrow range. The only way to have *CN*_2_ to be non-zero but different from *CN*_1_ is to be in the situation where the *CN*-nullcline for cell 2 is like the dashed blue line in Figure 4. In this case, the *ptc* threshold for *CN* production in cell 2 is fine-tuned to be very close to maximal *ptc* level. In a model with small Hill coefficients in the *CI-CN-PTC* sector, we would get *CN*_1_>*CN*_2_ and *ptc*_1_<*ptc*_2_ without such fine-tuning. We will come back to this point later.

We should point out that, in this study, we lay down the conditions only on the expression levels of key components *en*, *wg* and *hh* as specified in Equation 1. The reason, other than the simplicity of analysis, is that we believe the requirement of proper segment formation lays much stronger constraints on these key components compared to the rest. It is not clear to us that the *CI*-*CN*-*PTC* negative feedback has an extremely important role in segment formation stage of development. The study of von Dassow et al. [3] also uses a scoring function which rewards wild type levels only for these key components.

Having specified the requirements of functionality, let us now analyze what conditions are laid on the parameters of the model. Table 1 shows the set of inequalities characterizing the feasible region in the parameter space. Here we present the arguments leading to these conditions. The presence of *EN* in cell 3 requires the *WG* signaling for this cell to be above the activation threshold for *en*. This requirement is condition 3 in Table 1 (recall that *κ_XY_* can take value only between zero and one). Also, in this cell, *EN* will shut off the expression of *ci* (Equation 12) which is necessary for the production of *CI*, *ptc*, *PTC* and *PH*. Therefore, none of those components are expressed in cell 3. In cells 2 and 4, the expression level of these components has been shown to be the same (Equation 16). Therefore, we only need to focus on the expression of these components in cells 1 and 2.

Let 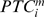 be the *PTC* level corresponding to the maximal *ptc* mRNA (*ptc* = 1) in cell *i*. If the threshold to produce *CN* is above 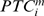, then cell *i* would not produce *CN*. As we pointed out before, the presence of *CN* in cells 1 and 2 is essential to repress *en* in those cells. These facts together necessitate condition 1 in Table 1.

**Figure 5:**
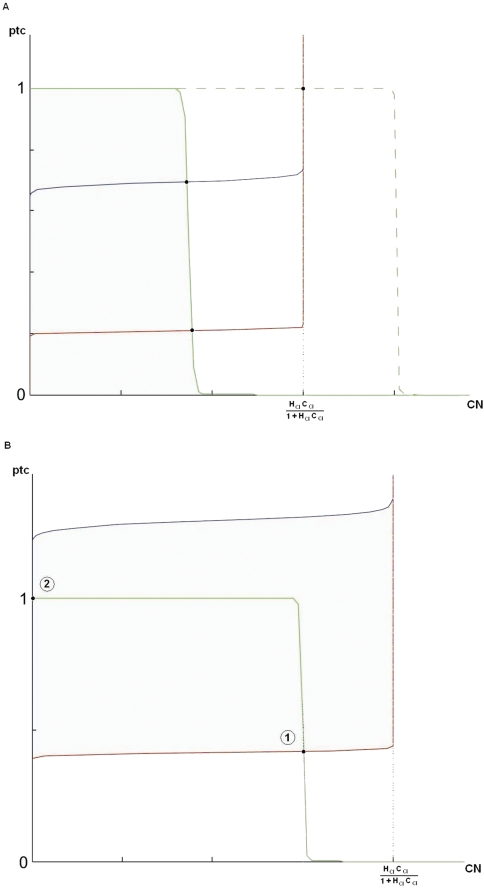
The nullclines for *ptc* and *CN*. (A) Blue and red curves show the *CN*-nullclines for relatively higher and lower values of *HH* signaling levels, respectively. The green curve shows the *ptc*-nullcline when 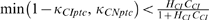. In this case, the negative feedback on *ptc* coming from repression by *CN* is active. Therefore, *ptc* and *CN* are not maximally expressed. The dashed green curve shows the other case where 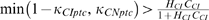. In this case, both *CN* and *ptc* are maximally expressed. This means that the negative feedback on *ptc* is inactive. (B) The green curve shows the *ptc*-nullcline. Blue and red curves show the *CN*-nullclines for relatively higher and lower values of *HH* signaling levels, respectively. The blue curve shows the situation where *HH* signaling is strong enough so that the *ptc* concentration needed to produce *CN* is higher than the maximal possible value for *ptc*, namely, one. Therefore, *CN* will not be produced in the corresponding cell. In the high Hill coefficient approximation, this is the only way that we can have *CN* level in cell 2 (intersection point 2) to be different from cell 1 (intersection point 1).

What would the *CN* level in cells 1 and 2 be when condition 1 is satisfied? As one sees from Figure 5A, there are two possibilities depending upon whether min(1−*κ_CIptc_*, *κ_CNptc_*) is smaller or larger than 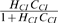. The case corresponding to *ptc*-nullcline in solid green has been discussed before. This is the case where *ptc* levels are affected by the negative feedback, and *CN* level is equal to min(1−*κ_CIptc_*, *κ_CNptc_*), which is less than its maximal possible value of 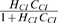. When the *ptc*-nullcline is like the dashed green line in Figure 5, *CN* levels in both cell 1 and cell 2 is equal to the maximal amount of 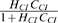, which is lower than min(1−*κ_CIptc_*, *κ_CNptc_*). In this case, the negative feedback is not active and *ptc* is maximally expressed (*ptc* = 1). We conclude that *CN* level is given by 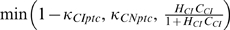, which we call *Z_C_*. We will now discuss the conditions to be satisfied by *Z_C_* for proper expression pattern of *en* and *wg*.

The *en* repression in cells 1 and 2 gives rise to condition 4 in Table 1. The fact that *CI*-*CN* pathway should not activate *wg* in cell 4 is guaranteed by condition 2 in Table 1. Consequently, *WG* in cell 2 has no contribution from *CI*-*CN* pathway (remember that cells 2 and 4 have the same *CI* and *CN* levels) and is solely produced by the autoactivation term. The autoactivation should only operate in cell 2 and nowhere else. This is condition 4 in Table 1.

von Dassow and Odell analyzed randomly generated solutions for the segment polarity model in reference [3] and plotted the marginal distribution of parameters (see Figure 6 of [16]). We can relate their results to the constraints presented in Table 1. From condition 1, we expect *κ_PTCCI_* to have tendency for lower values. From condition 2, we expect *κ_CNwg_* to have tendency for lower values and *κ_CIwg_* for higher values. Also, in order to have higher values for *Z_C_*, we expect *κ_CIptc_* to have tendency for lower values and *κ_CNptc_* for higher values. From condition 3 and 4, we expect *κ_EWGen_* and *κ_CNen_* to have tendency for lower values. From condition 5, we expect *κ_WGwg_* to have tendency for intermediate values. These expectations agree qualitatively with the results presented in Figure 6 of [16].

From Figure 6 of reference [16], we see that many of the parameters are uniformly distributed. One should note that a uniform distribution for a certain parameter could arise from two different scenarios. It could be the case that changing the parameter over a wide range of values does not influence the final outcome of the network. The other possibility is that the effect of changing the particular parameter could be compensated by changes in other parameters in such a way that for each value of the parameter, there is roughly equal number of solutions.

Now, let us contrast these set of conditions to the one obtained for the *SLP* model. Table 2 shows the conditions defining the feasible region for this case. For this regulatory network (Figure 2), instead of Equations 2 and 5, we have:
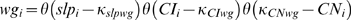
(21)

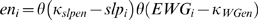
(22)


The rest of equations are the same as before (Equations 3, 4 and 6–14). Since *SLP* is present only in cells 1 and 2, *wg* has the possibility to be expressed only in those two cells. The decisive factor is *CN* levels in cells 1 and 2 (remember that, in these cells, *CI* = 1-*CN*). In the wild type pattern, *wg* is expressed only in cell 2 and this means that *CN* levels cannot be the same in cells 1 and 2. The only way to have less *CN* in cell 2 compared to cell 1 is to have 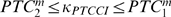. The condition 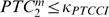 corresponds to the plateau in the *CN*-nullcline for cell 2 being higher or equal to the maximal *ptc* level (blue graph in Figure 5B). When it is higher, *CN*_2_ is zero and when it is fine-tuned to be equal, *CN*_2_ is between 0 and 1. If we had 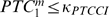, given that 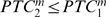, we would have *CN*_1_ = *CN*_2_ = 0. This is inconsistent with our requirement that *CN*_1_ and *CN*_2_ be different. Therefore, we have 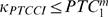. For our discussion, we will ignore the fine-tuned cases, leaving us with condition 1 in Table 2. This mean *CN*_2_ = 0 and 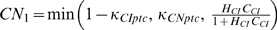 which we again call *Z_C_*. The condition 2 in Table 2 guarantees the absence of *wg* in cell 1. The fact that external *WG* signaling has to be strong enough in cell 3 to activate *en* but has to be weak enough in cell 4 not to produce *en* is coded in the condition 3 of Table 2.

### Random Walk in the Feasible Region

To get an estimate for the fractional volume of feasible region in the parameter space, we randomly chose 10^6^ parameter combinations and checked if they satisfy the conditions given in Tables 1 and 2 for the corresponding model. We perform the random walk by first selecting a random point, *P* ^0^, from the set of admissible parameters and follow successive random perturbations 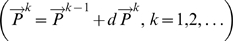. Each component of 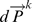 is selected from an independent Gaussian distribution with a standard deviation of 2*10^−3^. We follow this random walk until it hits a boundary and exits the space. This happens when one of the inequalities, which characterize the feasible region, is violated. Whenever the random walk exits the region, we record the time as well as the condition that was violated and therefore caused the exit. The parameter ranges were similar to those used in [3], except that we facilitated the transport processes for *hh* and *PTC*. We simulated the random walk for 30,000 runs.

## Acknowledgments

AMS thanks Pankaj Mehta for discussions that lead to the formulation of the high Hill coefficient version of the segment polarity network model. We also thank Viji Nagaraj for carefully reading the final manuscript.

## References

[1] Alon U, Surette MG, Barkai N, Leibler S (1999). Robustness in bacterial chemotaxis.. Nature.

[2] Little JW, Shepley DP, Wert DW (1999). Robustness of a gene regulatory circuit.. EMBO J.

[3] von Dassow G, Meir E, Munro E, Odell G (2000). The segment polarity network is a robust developmental module.. Nature.

[4] Savageau M (1971). Parameter sensitivity as a criterion for evaluating and comparing the performance of biochemical systems.. Nature.

[5] Heinrich R, Schuster S (1996). The Regulation of Cellular Systems.

[6] Brown KS, Sethna JP (2003). Statistical mechanical approaches to models with many poorly known parameters.. Phys Rev E Stat Nonlin Soft Matter Phys.

[7] Brown KS, Hill CC, Calero GA, Myers CR, Lee KH (2004). The statistical mechanics of complex signaling networks: nerve growth factor signaling.. Phys Biol.

[8] Gutenkunst RN, Waterfall JJ, Casey FP, Brown KS, Myers CR (2007). Universally sloppy parameter sensitivities in systems biology models.. PLoS Comput Biol.

[9] Ingolia N (2004). Topology and robustness in the Drosophila segment polarity network.. PLoS Biol.

[10] Albert R, Othmer H (2003). The topology of the regulatory interactions predicts the expression pattern of the Drosophila segment polarity genes.. J Theor Biol.

[11] Waxman D, Gavrilets S (2005). 20 questions on adaptive dynamics.. J Evol Biol.

[12] Sengupta AM, Djordjevic M, Shraiman BI (2002). Specificity and robustness in transcription control networks.. Proc Natl Acad Sci U S A.

[13] Chaves M, Albert R, Sontag E (2005). Robustness and fragility of Boolean models for genetic regulatory networks.. J Theor Biol.

[14] Chaves M, Sontag E, Albert R (2006). Methods of robustness analysis for Boolean models of gene control networks.. IEE Proc Syst Biol.

[15] Ma W, Lai L, Ouyang Q, Tang C (2006). Robustness and modular design of the drosophila segment polarity network.. Mol Syst Biol.

[16] von Dassow G, Odell G (2002). Design and constraints of the drosophila segment polarity modude: robust spatial patterning emerges from intertwined cell state switches.. J Exp Zool.

[17] Cadigan K, Grossniklaus U, Gehring W (1994). Localized expression of sloppy paired protein maintains the polarity of Drosophila parasegments.. Genes Dev.

[18] Tenenbaum JB, de Silva V, Langford JC (2000). A global geometric framework for nonlinear dimensionality reduction.. Science.

[19] Roweis ST, Saul LK (2000). Nonlinear dimensionality reduction by locally linear embedding.. Science.

[20] Hidalgo A, Ingham P (1990). Cell patterning in the Drosophila segment: spatial regulation of the segment polarity gene patched.. Development.

